# Leveraging Multi-Annotator Label Uncertainties as Privileged Information for Acute Respiratory Distress Syndrome Detection in Chest X-ray Images

**DOI:** 10.3390/bioengineering11020133

**Published:** 2024-01-29

**Authors:** Zijun Gao, Emily Wittrup, Kayvan Najarian

**Affiliations:** 1 Department of Computational Medicine and Bioinformatics, University of Michigan, Ann Arbor, MI 48109, USA; ewittrup@med.umich.edu (E.W.); kayvan@med.umich.edu (K.N.); 2 Michigan Institute for Data Science (MIDAS), University of Michigan, Ann Arbor, MI 48109, USA; 3 Department of Emergency Medicine, University of Michigan, Ann Arbor, MI 48109, USA; 4 Max Harry Weil Institute for Critical Care Research and Innovation, University of Michigan, Ann Arbor, MI 48109, USA

**Keywords:** acute respiratory distress syndrome, chest X-ray, learning using privileged information, label uncertainty, label noise

## Abstract

Acute Respiratory Distress Syndrome (ARDS) is a life-threatening lung injury for which early diagnosis and evidence-based treatment can improve patient outcomes. Chest X-rays (CXRs) play a crucial role in the identification of ARDS; however, their interpretation can be difficult due to non-specific radiological features, uncertainty in disease staging, and inter-rater variability among clinical experts, thus leading to prominent label noise issues. To address these challenges, this study proposes a novel approach that leverages label uncertainty from multiple annotators to enhance ARDS detection in CXR images. Label uncertainty information is encoded and supplied to the model as privileged information, a form of information exclusively available during the training stage and not during inference. By incorporating the Transfer and Marginalized (TRAM) network and effective knowledge transfer mechanisms, the detection model achieved a mean testing AUROC of 0.850, an AUPRC of 0.868, and an F1 score of 0.797. After removing equivocal testing cases, the model attained an AUROC of 0.973, an AUPRC of 0.971, and an F1 score of 0.921. As a new approach to addressing label noise in medical image analysis, the proposed model has shown superiority compared to the original TRAM, Confusion Estimation, and mean-aggregated label training. The overall findings highlight the effectiveness of the proposed methods in addressing label noise in CXRs for ARDS detection, with potential for use in other medical imaging domains that encounter similar challenges.

## 1. Introduction

Acute Respiratory Distress Syndrome (ARDS) is an inflammatory lung injury characterized by diffused alveolar damage. It occurs in critically ill patients due to various etiologies such as major trauma, pneumonia, and sepsis. As a prevalent medical condition worldwide, ARDS affects over 3 million people of all ages annually [1]. Due to the nonspecific manifestations of ARDS, it can easily go unrecognized in patients until it is severe [2], which leads to a hospital mortality rate of approximately 45% [2,3].

Chest X-rays (CXRs) are key in the clinical diagnosis of ARDS, specifically the radiological presence of bilateral infiltrates. CXRs usually demonstrate evidence of ARDS in the form of bilateral diffuse alveolar opacities, which may appear as consolidations as ARDS progresses. Nevertheless, the image findings may vary depending on the stage and severity of ARDS, and they may be subtle within the first 24 h following lung insult [4]. Additionally, radiological features alone are nonspecific and may not correlate with clinical findings. As a result, poor agreements (a Cohen’s κ of <0.27) among clinicians on CXR interpretations for ARDS diagnosis have been reported [5,6]. Given that ARDS has a fast-progressing nature, recognizing and treating this condition promptly is crucial for better patient outcomes. Therefore, approaches that can identify ARDS from CXRs are urgently needed to provide patients with timely and evidence-based care.

Previous studies have used traditional machine learning (ML) and deep learning (DL) approaches to detect ARDS from CXRs. Zaglam et al. [7] considered the image texture of intercostal patches for distinguishing between CXRs with ARDS and those without. After identifying the patches by the semiautomatic segmentation of ribs, histogram features, co-occurrence matrix features, and spectral features were obtained and fed into a support vector machine (SVM) for classification. Reamaroon et al. [8] employed SVM, random forest, and tree-based boosting classifiers to detect ARDS based on handcrafted features extracted from entire CXRs. These features included directional blur features that capture the cloudiness in the CXR, histogram features, co-occurrence matrix features, and features from pre-trained deep neural networks. Regarding DL approaches, Sjoding et al. [9] proposed an automatic ARDS detection network with Densenet [10], a widely used architecture for medical imaging analysis. They first pre-trained the network by supervised learning on public datasets, then fine tuned it with ARDS images for a downstream classification task. In addition, they utilized GRAD-Cam to highlight the potential ARDS findings on CXRs through saliency maps. On the other hand, Yahyatabar et al. [11] developed the Dense-Ynet model for stratifying the severity of ARDS in CXR images by performing the segmentation and classification tasks simultaneously. A global ARDS severity score for the CXRs was provided based on the distribution of infiltrates in different lung quadrants.

Despite the effectiveness of existing approaches, previous research has not adequately addressed concerns related to label uncertainty and noise. This is particularly notable given the substantial inter-reviewer variability and poor agreements in ARDS diagnosis. For example, Yahyatabar et al. [11] chose to exclude images with labeling disagreements. Similarly, in studies by Reamaroon et al. [8] and Sjoding et al. [9], although uncertain annotations from multiple clinicians were present in the dataset, the training and validation labels relied solely on mean-aggregated values. This approach potentially exposes the model to challenges in the performance and generalizability arising from noisy labels.

In the field of deep learning for medical image analysis, several strategies [12] have been proposed to address the challenge of label noise, including label smoothing [13], network structure modification [14], and data reweighting [15,16]. However, none of the existing approaches have fully utilized label uncertainty from multiple experts, and the prevailing practice of label averaging persists when multiple annotations are available. Notably, two studies, namely Confusion Estimation [17] and the Transfer and Marginalize (TRAM) network [18], have emerged as promising solutions to this challenge. Confusion Estimation addresses observer confusion by simultaneously estimating correct labels and annotator confusion matrices during network training. This method has demonstrated significant improvements in tasks such as natural image classification and ultrasound cardiac view classification, which is a medical imaging task. On the other hand, the TRAM network incorporates the annotator’s information as privileged information, which is available only during training and not during inference [19]. By employing a two-branch network architecture consisting of the base and privileged branches, as well as updating the base feature extractor solely through the privileged branch during training, the TRAM network encourages the inclusion of knowledge from the privileged branch in the base branch during testing when the privileged branch is no longer needed. In [18], the privileged branch utilized multi-annotator labels and one-hot encoded annotator IDs as privileged information, leading to enhanced performance in natural image tasks. However, its applicability to medical data has not been investigated.

In this paper, we present a novel deep-learning model inspired by the TRAM [18] method to enhance the detection of ARDS in CXR images by leveraging label uncertainty from multiple annotators as privileged information. We propose three distinct encoding methods and a simple yet effective measurement of uncertainty. By incorporating a mechanism to provide the model with privileged information only when necessary and refining the privileged branch to apply ordinal regression on its output, the proposed model facilitates more effective knowledge transfer from the privileged branch to the base branch. As a result, the model achieves superior testing performance compared to the original TRAM, Confusion Estimation, and other baseline models trained with mean-aggregated labels.

The main contributions of this work can be summarized as follows:

1. We proposed a novel deep learning model that leverages label uncertainty from multiple annotators to enhance the discriminative performance in identifying ARDS from CXR images.
2. We introduced effective encoding methods and a measure of uncertainty for handling multi-annotator uncertain labels.
3. We enhanced the TRAM network to address the label noise issue in medical image analysis by incorporating specially designed mechanisms that facilitate effective knowledge transfer. The proposed enhancements can be extend to other medical imaging tasks and various computer vision applications.

Overall, the significance of this work lies in its approach in tackling the label noise arising from multi-annotator label uncertainty in medical image analysis, particularly in the context of ARDS detection in CXR images. On the one hand, it has the potential to mitigate the impact of label noise and improve the accuracy of ARDS detection, which can ultimately lead to more effective diagnoses and treatments of this life-threatening condition. On the other hand, this methodology can be extended to address the analogous challenges encountered in other medical imaging problems.

The remainder of this manuscript unfolds as follows. Section 2 introduces the dataset used in this study and delves into the encoding methods and measures of uncertainty. It provides further details on the model implementation, experimental setup, training strategy, test metrics, and qualitative evaluation. Moving on, Section 3.1 presents the test performances of the models on all test cases or stratified test cases. In Section 4, we provide interpretations of the results and discuss the limitations of the current work, as well as offer potential directions for future research.

## 2. Materials and Methods

### 2.1. Dataset

#### 2.1.1. Inclusion Criteria

The study cohort was formed by retrospectively identifying adult patients admitted to intensive care units at Michigan Medicine from 2016 to 2017 who met either of the following criteria: (1) acute hypoxic respiratory failure, as defined by a PaO_2_/FiO_2_ ratio of <300 mm Hg while receiving invasive mechanical ventilation, or (2) moderate hypoxia, which requires more than 3 L of supplemental oxygen via the nasal cannula for at least 2 h.

These inclusion criteria were designed to encompass a diverse patient population that is representative of real-world clinical settings, which included patients with potential lung disease phenotypes other than ARDS. As such, the objective of the study was to accurately identify ARDS in patients presenting with a range of respiratory illnesses, rather than differentiate between healthy and ARDS patients.

#### 2.1.2. Characteristics

**Figure 1:**
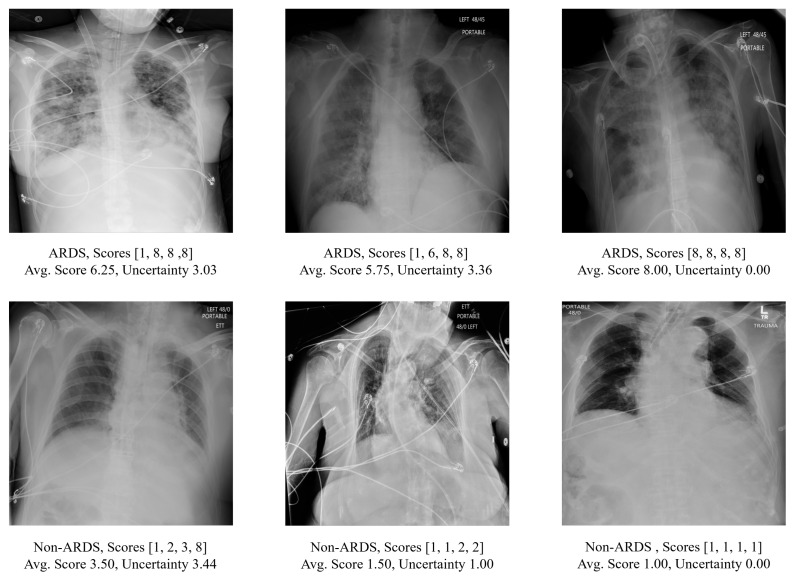
The upper panel displays the CXR scans of patients diagnosed with ARDS, while the lower panel shows scans of patients without ARDS. The score array represents the annotation score provided by multiple reviewers together with the averaged score and the corresponding measurement of uncertainty (as defined in Section 2.3).

Examples of CXRs in this dataset are shown in Figure 1. Since the CXRs were obtained from hospitalized settings, they exhibited a wide range of variations and complexities. These include variations in image quality such as dynamic range and sharpness, the presence of medical devices or implants, and the manifestation of the disease itself. In total, the cohort consisted of 3055 anteroposterior (AP) CXRs from 500 patients. As depicted in Table 1, 2050 CXRs from 333 patients admitted in 2016 were used in training, while 1005 CXRs from 167 patients admitted in 2017 were designated as the hold-out test set, with no patient overlap in the data split. Among these 500 patients, 309 were male and 191 were female. The average age of the patients was 57.65 years, with a standard deviation of 16.32 years. Further information regarding patient demographics can be found in Table 2.

#### 2.1.3. Label Scheme

**Figure 2:**
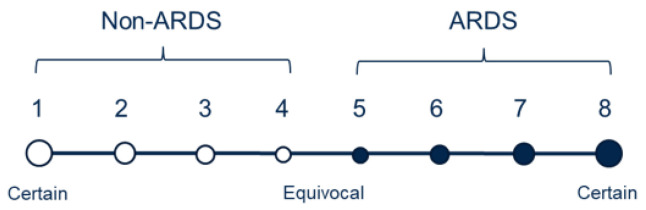
Diagram for the different labeling scores on a scale of 1 to 8. Solid circles indicate diagnoses of ARDS, while empty ones represent non-ARDS. The size of the circles represents the certainty level of an assigned score.

Fourteen physicians trained in critical care medicine independently evaluated the CXRs, with each image receiving two to four evaluations. The evaluations primarily relied on the presence of bilateral opacities, which were supplemented by reviewing other clinical information during the patient’s hospitalization. As illustrated in Figure 2, the physicians used an ordinal scale ranging from 1 to 8 to rate the presence of ARDS, with a rating of 1 indicating high confidence that the CXR did not show ARDS, a rating of 8 indicating high confidence of ARDS presence, and a rating of 4 or 5 indicating equivocal findings. Detailed information regarding the distribution of the number of reviewers per image and the total images reviewed per reviewer can be found in Appendix A.

#### 2.1.4. Label Agreement

**Figure 3:**
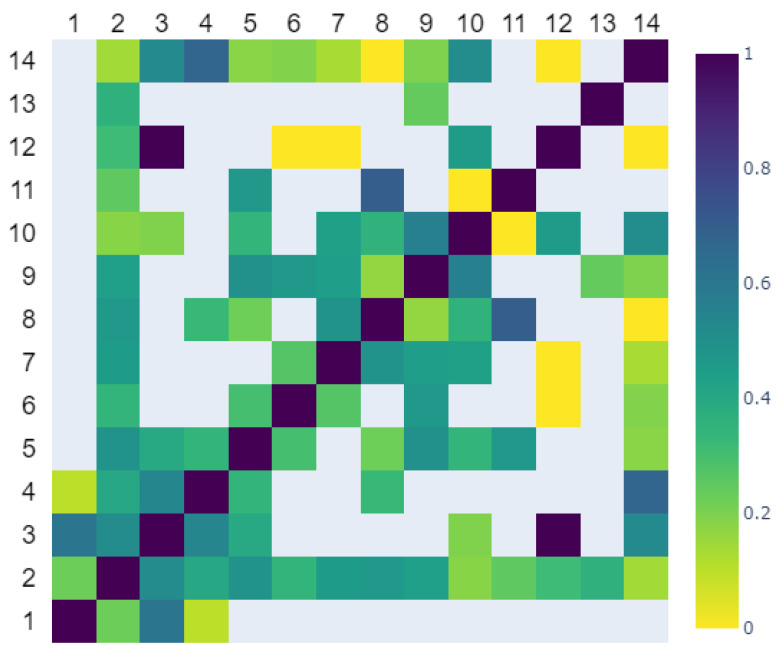
The Cohen Kappa scores between the pairs of 14 independent reviewers’ agreement of ARDS diagnosis from the CXR images.

To assess the agreement in the labeling among different reviewers, the evaluations provided by each reviewer were binarized by applying a threshold of 4.5 to the annotated scale. A Cohen’s κ coefficient was subsequently computed between each pair of the reviewers based on the images that were reviewed by both reviewers. In cases where there were no shared images for a specific pair of reviewers, the resulting κ value was set as NaN (Not a Number). The mean Cohen’s κ value was around 0.366, indicating only a fair level of agreement between reviewers [20]. Figure 3 displays a heatmap depicting the pairwise Cohen’s κ values among the 14 reviewers.

#### 2.1.5. Mean Label Aggregation

The CXR labels, *y*, were determined by averaging the annotated scores that were assigned by different physicians. If the average score was below 4.5, the CXR was labeled as non-ARDS; otherwise, it was labeled as ARDS. By employing this approach, a total of 933 CXR images were identified as meeting the criteria for ARDS, while 2122 images were labeled as non-ARDS. As listed in Table 1, there were 606 ARDS CXR images and 1444 non-ARDS images within the training set. In the holdout test set, the numbers stood at 327 ARDS CXR images and 687 non-ARDS images. However, due to the high level of label disagreements among the physicians, this approach inherently introduced noisy labels. In the following sections, methods will be introduced to measure the uncertain levels associated with these labels and to provide a more reliable assessment of the labels during the testing phase.

### 2.2. Encoding of Multi-Annotator Information

Assuming there are *k* annotations on a CXR image *x*, where k∈{2,3,4}, each annotation is represented by an annotation score $S_{i}\in S=\left\{1,2,\ldots ,8\right\}$, and it is also associated with a reviewer’s ID, as denoted by $T_{i}\in T=\left\{1,2,3,\ldots ,14\right\}$. In this context, a sequence $\left\{\left(S_{1},T_{1}\right),\ldots ,\left(S_{k},T_{k}\right)\right\}$ corresponds to the annotation scores provided by *k* reviewers, with each $S_{i}$ linked to the respective reviewer’s ID as $T_{i}$. To illustrate this, consider the sequence {(6,8),(2,6),(2,12)}, which represents three reviewers with reviewer IDs 8, 6, and 12, as well as corresponding annotation scores of 6, 2, and 2, respectively. To incorporate this information into the training of our proposed methods, we introduced three encoding protocols as follows:

- **Score Encoding** (Score. E.): Only the annotation score is encoded. The encoder vector is represented as **E** $=\left[E_{1},E_{2},\ldots ,E_{7},E_{8}\right]\in R^{8}$, where each element is
  $$\begin{array}{cc} E_{s} & =\sum_{i=1}^{k}\left\{\begin{array}{cc} 1, & \mathrm{if}\;S_{i}=s\\ 0, & \mathrm{otherwise} \end{array}\right.,\;s\in S. \end{array}$$
  The value of $E_{s}$ represents the count of occurrence of the corresponding score value *s* among the *k* annotations. S represents the set of all possible scores.
- **Separate Encoding** (Separ. E.): Both the annotation scores and the annotator IDs are encoded. The encoder vector for annotation scores is the same as that in the Score Encoding protocol, while the one for the annotator ID is represented as $A=\left[A_{1},A_{2},\ldots ,A_{13},A_{14}\right]\in R^{14}$, where
  $$\begin{array}{cc} A_{t} & =\left\{\begin{array}{cc} 1, & \mathrm{if}\;t\in \{T_{1},\ldots ,T_{K}\}\\ 0, & \mathrm{otherwise} \end{array}\right.,\;t\in T, \end{array}$$
  and T represents the set of all possible annotator IDs. A and E are then concatenated to form the final encoder vector in $R^{22}$.
- **Combine Encoding** (Comb. E.): Both the annotation score and the annotator ID are encoded. The encoder vector is $C=\left[C_{1},C_{2},\ldots ,C_{13},C_{14}\right]\in R^{14}$. If an annotation $T_{i}$ is provided by annotator *t* with score $S_{i}$, then $C_{t}$ takes the value of $S_{i}$. Otherwise, it is assigned a value of 0. The formulation is
  $$\begin{array}{cc} C_{t} & =\left\{\begin{array}{cc} S_{i}, & \mathrm{if}\;t\in \{T_{1},\ldots ,T_{K}\}\\ 0, & \mathrm{otherwise} \end{array}\right.,\;t\in T. \end{array}$$

### 2.3. Measure for Uncertainty

The uncertainty of an ARDS diagnosis from a CXR image arises from two sources: the annotation score provided by a reviewer and the agreements or disagreements among reviewers. As discussed earlier, a rating of 1 or 8 indicates a higher certainty from the physician regarding the presence or absence of ARDS findings in the CXR. However, uncertainty is not solely dependent on the annotation score, but also on the level of agreement between reviewers. Higher reviewer disagreements generally indicate a higher level of uncertainty for a given case.

Therefore, following the notions described in the previous section, we designed the following measure of uncertainty:

$$\begin{array}{c} D=\frac{1}{k}\sum_{i}^{K}g\left(S_{i}\right)+\sigma_{S_{1},\ldots ,S_{k}}. \end{array}$$

This was used to quantify the uncertainty at the image level, where $\sigma_{S_{1},\ldots ,S_{k}}$ is the standard deviation component that takes into account the variability in the scores $S_{i}$ assigned by different reviewers and the function g(s):S→R, which is defined as follows:

$$\begin{array}{c} g\left(s\right)=-\left|s-4.5\right|+3.5,s\in S. \end{array}$$

The function g(s) captures the degree of uncertainty associated with each annotation score. It assigns lower values of uncertainty to high-confidence ratings of 1 or 8 and a higher value of uncertainty for equivocal ratings such as 4 or 5.

Table 3 presents the summary statistics of *D* on the training and testing sets, thereby providing a comprehensive overview of its distribution and variability. In the training set, the mean measurement of uncertainty was 1.95 with a standard deviation of 1.29, ranging from a minimum of 0.00 to a maximum of 3.92. The 25th percentile (Q1) was 1.00, the median was 2.00, and the 75th percentile (Q3) was 3.25. Similarly, the mean measurement of uncertainty in the testing set was 1.86 with a standard deviation of 1.23, ranging from 0.00 to 3.83. The 25th percentile, median, and 75th percentile values were consistent with those of the training set.

Although *D* may not serve as an unbiased estimator as in [16,21], it was proved to be effective when combined with the thresholding mechanism described in Section 2.6. This combination successfully promoted knowledge transfer in the proposed models.

### 2.4. Supervised Per-Trained Encoder

The encoder used in this study was a ResNet50 [22] model that was pretrained using supervised learning and the weight was obtained from the TorchXrayVision (https://github.com/mlmed/torchxrayvision/ (accessed on 23 April 2023)) repository [23]. By leveraging the knowledge learned from diverse publicly available datasets, including the RSNA Pneumonia Challenge (https://www.kaggle.com/c/rsna-pneumonia-detection-challenge (accessed on 23 April 2023)), NIH Chest X-ray8 [24], PadChest [25], CheXpert [26], and MIMIC-CXR datasets [27], the pretrained ResNet50 encoder provided a strong foundation for our model to extract meaningful features from the CXR images.

To ensure compatibility with the pretrained encoder, the CXR images were processed in accordance with [23]. They were resized to a dimension of 512 × 512 and then normalized to a range of [−1024, 1024].

### 2.5. Proposed Method

**Figure 4:**
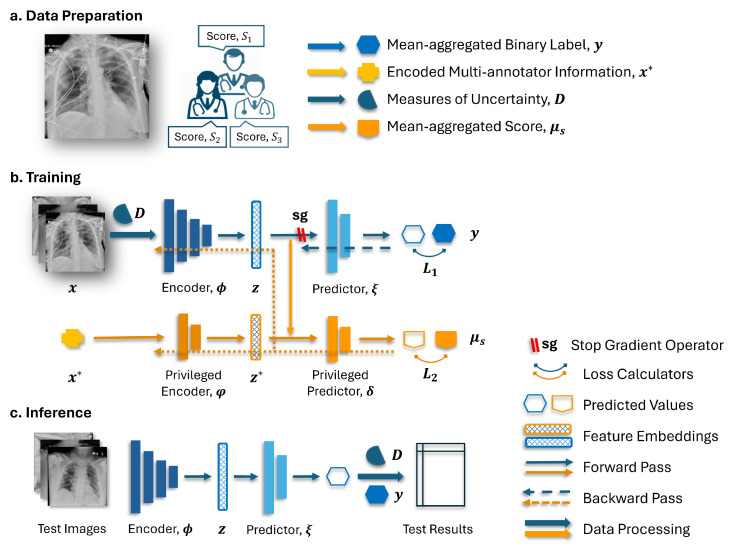
Schematic diagram for (**a**) the data preparation, (**b**) training, and (**c**) inference pipelines.

Figure 4 illustrates the diagram of the proposed method encompassing data preparation, model training, and inference. As depicted in Figure 4b, the employed model integrated two branches in its network architecture. The base branch includes an encoder denoted as ϕ and a predictor labeled as ξ. Meanwhile, the privileged branch comprised a privileged encoder represented as φ and a privileged predictor labeled as δ.

In the training stage, the encoder ϕ(x) generated an embedding from the input CXR images *x*. The resulting embedding, denoted as *z*, serves two purposes. Firstly, it is passed to the predictor ξ(z) on the base branch for the primary task. Secondly, it is concatenated with the privileged annotation information encoded as $z^{*}=\varphi \left(x^{*}\right)$ and utilized by the predictor $\delta (z^{*},z)$ on the privileged branch. With a stop gradient (sg) operator applied to the base branch, the back-propagation on the encoder ϕ only occurs through the privileged branch. This mechanism, initially introduced in [18] and named TRAM, enables the exploitation of the privileged annotation information $x^{*}$ to enhance the learning process of the base branch. During the testing phase, only the base branch was retained for making predictions.

The TRAM mechanism may encounter limitations when the annotation information obtained from the privileged branch contains excessive details about the target label [28]. This situation arises as the model may heavily rely on the embedded score annotations $z^{*}$ in conjunction with $\delta (z^{*},z)$ on the privileged branch, thus overshadowing the importance of the learning associations between the input data *x* and the target label through the encoder ϕ. Consequently, the models may struggle to generalize well to unseen examples or to exhibit limited performance in the testing phase, where privileged information is not available. Therefore, it is crucial to strike a balance in utilizing the privileged information while ensuring that the base branch also learns from the input data to obtain an encoder that produces robust and meaningful representations.

Two strategies were proposed to address the aforementioned issue. Firstly, the model was provided with the privileged annotation, which was encoded as described in Section 2.2, albeit only when the measurement of uncertainty, which was introduced in Section 2.3, exceeded a certain threshold. When the uncertainty fell below this threshold, an all-zero vector was used as a substitute for the privileged annotation information. By employing this strategy, the model was encouraged to utilize the multi-annotator privileged information primarily in the cases where the label may be noisy while also promoting the learning of associations between clean samples and their corresponding labels within the encoder. Secondly, instead of using binarized labels as the prediction target for the privileged branch, a rank-consistent ordinal prediction approach was employed, where the averaged scores $\mu_{S}$ among the annotators were rounded up and used as targets. This approach created a more nuanced prediction target that effectively captured the ordinal nature of the labels. Together with the thresholding mechanism, it will further encourage the learning of the clean instances during network training and help with knowledge transfer across branches.

### 2.6. Implementation Details and Training Logic

The models described below were implemented using PyTorch 1.10 and Python 3.7. The experiments were conducted on two Tesla V100 GPUs, each equipped with 16 GB of memory.

In our proposed model, the encoder ϕ is a ResNet50 model (as described in Section 2.4) with its final prediction layer removed. The resulting embeddings have a dimension of 2048. For the predictor ξ, we employed a linear layer to map the embeddings to the 2-dimensional output. On the privileged branch, the privileged encoder φ was implemented as a linear layer with 64 units, which was followed by batch normalization and ReLU activation to facilitate effective information flow and non-linearity. The output of φ was then concatenated with the embeddings *z* and passed into the privileged predictor, which was a network consisting of two layers. Each layer had 128 units, with batch normalization and ReLU activation applied between the layers. Notably, the final layer of the privileged predictor was specifically modified to align with a rank-consistent ordinal regression framework known as CORN [29]. This choice was underpinned by its demonstrated efficiency across various datasets [29] and its suggested superior performance, which was coupled with fewer structural constraints during training compared to alternative rank-consistent losses like CORAL [30].

The loss function L for training the network was defined as follows:

$$L=L_{1}\left(\xi \left[\mathrm{sg}(\phi (x))\right],y\right)+\beta L_{2}\left(\delta \left[\varphi \left(x^{*}\right),\phi \left(x\right)\right],\mu_{S}\right).$$

Here, $L_{1}$ represents the cross-entropy loss on the base branch, which measures the discrepancy between the predicted label distribution and the mean aggregated labels *y*. The function sg(·) denotes the stop gradient operation applied to the output of the encoder ϕ(x), thus ensuring that no gradients flow through the base branch during back-propagation. On the other hand, $L_{2}$ represents the CORN loss [29] on the privileged branch. The target values $\mu_{S}\in S$ were obtained by averaging the scores provided by multiple annotators and rounding them to the closest integer. The weight parameter β determines the relative significance of the privileged branch loss compared to the base branch loss. In the experiments, β was consistently set to 0.5, which was achieved by considering that the search for the learning rate could adequately incorporate the impact of both losses, as mentioned in [18].

In addition, the following models were implemented to provide a basis for comparison. The encoder architectures in these models remained unchanged from the previous description. In addition, the three encoding methods were independently applied in experiments for models that require annotator information encoding to evaluate the effectiveness.

1. **Linear Probing**: The encoder is frozen, and the predictor ξ is a linear layer with an input feature size of 2048 and an output dimension of 2. The objective is to minimize the cross-entropy loss between the predicted label distribution and the mean-aggregated labels.
2. **Fine Tuning**: The predictor architecture and the loss remain the same as in the Linear Probing architecture but with a trainable encoder.
3. **Confusion Estimation**: This model follows the same architecture as the Linear Probing approach but introduces trainable confusion matrices specific to each of the 14 reviewers. The prediction targets are obtained by binarizing the scores provided by each reviewer, using a threshold of 4.5. Other details follow the approach outlined in [17].
4. **TRAM**: This architecture is identical to the proposed model except that no thresholding (Thresh.) is applied when supplying privileged annotation information and no ordinal regression (Ord.Reg.) is used in the privileged branch.
5. **TRAM w/Thresh.**: This model builds upon the TRAM framework but incorporates the thresholding mechanism, where a threshold is applied to determine whether to use privileged annotation information.
6. **TRAM w/Ord. Reg.**: This model builds upon the TRAM framework but has the privileged branch that uses an ordinal regression in training.

The Adam optimizer [31] with default parameters was used in all the conducted experiments. The hyperparameters of interest were the learning rates for the encoder, denoted as α, and the learning rate β for the rest of the network. To determine the optimal learning rates, a grid search was performed over the values α∈{1e−4,4e−4,1e−5,5e−5,1e−6} and β∈{1e−3,5e−3,1e−4,5e−4,1e−5}. To ensure consistency in comparing the encoding methods and to avoid extensive hyperparameter tuning, the threshold level that distinguishes between more uncertain and less uncertain cases was not considered a hyperparameter in our experiment. Instead, the median value of 2 was selected as the threshold based on the statistics provided in Table 3. However, the choice of threshold level did have an impact on the performance of the proposed models. As the threshold increased from 0 to its maximum, the validation and testing performance initially improved and then started to decline. Details on the influence of applying different thresholds to validation and testing outcomes are listed in Appendix B.

Two separate random seeds were utilized to carry out the experiments. The first seed was employed for hyperparameter selection, where the model was trained using a three-fold cross-validation on the training set. The data splits were performed in a patient-wise manner. Each fold was trained up to 40 epochs using a batch size of 64, and early stopping was triggered if the validation loss did not decrease for 10 consecutive epochs. Among the models trained on each fold, the one with the lowest validation loss was identified as the optimal model. By calculating the mean statistics of the validation loss across the optimal models from all three folds, we were able to determine the optimal combination of hyperparameters. The second seed was used to repeat the three-fold cross-validation process with the optimal hyperparameters. The optimal models obtained from each fold were applied to the holdout test set, and the mean test metrics and standard deviation were reported.

Furthermore, while the training loss, target label, and network architecture may differ among different methods, the validation process was consistently conducted on the same architecture depicted in Figure 4c. This architecture utilized mean-aggregated labels and cross-entropy loss. To ensure the reliability of the validation set within each fold and prevent potential misleading results, only cases with an uncertain level of 2 or lower were included in the validation set after their assignment during cross-validation. This filtering process ensured that the validation set consisted of cases with relatively low uncertainty levels.

### 2.7. Test Evaluation

The performance metrics involved in model evaluation are precision, accuracy, sensitivity, specificity, F1 score, the Matthews correlation coefficients (MCC) [32], the area under the receiver operating characteristic curve (AUROC), and the area under the precision–recall curve (AUPRC). The first six metrics were defined as

$$\begin{array}{cc} \mathrm{Precision} & =\frac{TP}{\left(TP+FP\right)},\\ \mathrm{Accuracy} & =\frac{\left(TP+TN\right)}{\left(TP+TN+FP+FN\right)},\\ \mathrm{Sensitivity} & =\frac{TP}{TP+FN},\\ \mathrm{Specificity} & =\frac{TN}{TN+FP},\\ F1\;\mathrm{Score} & =\frac{2\times \mathrm{Sensitivity}\times \mathrm{Precision}}{\mathrm{Sensitivity}+\mathrm{Precision}},\\ \mathrm{MCC} & =\frac{\mathrm{TP}\times \mathrm{TN}-\mathrm{FP}\times \mathrm{FN}}{\sqrt{\left(\mathrm{TP}+\mathrm{FP})(\mathrm{TP}+\mathrm{FN})(\mathrm{TN}+\mathrm{FP})(\mathrm{TN}+\mathrm{FN}\right)}} \end{array}$$

where *TP, TN, FP*, and *FN* are the count of true positives, true negatives, false positives, and false negatives samples, respectively.

Due to the absence of gold standard labels for the test set, we employed two evaluation approaches. The first approach utilized mean-aggregated labels, while the second approach categorized the predictions based on their uncertainty into two distinct ranges, i.e., [0, 2) and [2, 4), which allowed us to analyze the model’s performance over different levels of uncertainty. We paid more attention to cases with lower measurements of uncertainty as they were more likely to have accurate labels.

### 2.8. Visual Explanations

To generate visual explanations for the best-performing proposed models, we utilized ScoreCAM [33], a CAM-based visualization method that offers post hoc visual explanations, on the test images. The implementation (https://github.com/jacobgil/pytorch-grad-cam (accessed on 3 January 2024)) from [34] was employed, with layer 4 in the ResNet50 designated as the target layer.

## 3. Results

### 3.1. Performance Analysis of the Baseline and Proposed Models on the Test Set with Mean Aggregated Labels

Table 4 presents the testing statistics across the different models, and it also shows the comparisons of the model predictions against the mean-aggregated labels. The upper panel (Table 4a) showcases the baseline models’ performances, while the lower panel (Table 4b) focuses on the results for the proposed models. The best-performing metrics are highlighted in bold for each tested metric in their respective panels for easier comparison. The model names and encoding methods correspond to the abbreviations detailed in Section 2.2 and Section 2.6.

Among the tested baselines in Table 4a, the TRAM models with the threshold mechanism (w/Thresh) and the Separate Encoding approach outperformed other models across almost all metrics. It achieved a precision of 0.792, an accuracy of 0.796, an AUROC of 0.872, a sensitivity of 0.802, a specificity of 0.789, an F1 score of 0.797, and an MCC of 0.591. The Confusion Estimation model attained the highest AUPRC of 0.870. When comparing the different groups of TRAM-based models, it was notable that the inclusion of the thresholding mechanism had a significant impact. The TRAM w/Thresh models, when compared to the original TRAM models, demonstrated a 2–3% increase across all metrics. Disregarding this mechanism can have a detrimental effect, as further illustrated by the fact that the original TRAM model performed unfavorably on most metrics when compared to the Linear Probing and Fine Tuning approaches.

Moreover, the incorporation of ordinal regression can lead to performance improvements ranging from 1% to 3% compared to the original TRAM models, thus helping to mitigate the performance drop in the absence of the thresholding mechanism. Additionally, the performance of the Linear Probing approach was found to be comparable to that of the Fine Tuning method, with only a slight difference observed in the AUPRC. This indicated that the supervised pre-trained encoder is capable of capturing meaningful embeddings from the CXR images. Furthermore, it suggests that the Fine Tuning approach could potentially overfit the training data, thus resulting in similar performances to the Linear Probing method despite having higher learning powers.

Among the proposed models in Table 4b, the best results were achieved when using the proposed model with a Score Encoding methodology. This approach yielded a precision of 0.798, an accuracy of 0.797, an AUPRC of 0.868, an AUROC of 0.873, a sensitivity of 0.796, a specificity of 0.798, an F1 score of 0.797, and an MCC of 0.594. Furthermore, compared to the TRAM models that incorporated ordinal regression on the privileged branch, as shown in Table 4a, the proposed models that additionally utilized the thresholding mechanism demonstrated a performance improvement of up to 1% in all the testing metrics. This finding reinforces the importance of the thresholding mechanism in achieving effective models.

In summary, among all the tested models, the proposed network with a Score Encoding method achieved the highest precision, accuracy, AUROC, specificity, F1 score, and MCC. Conversely, the TRAM model when utilizing thresholding and the Separate Encoding approach attained the highest sensitivity, while the Confusion Estimation model yielded the highest AUPRC. The incorporation of a thresholding mechanism and the utilization of ordinal regression on the privileged branch were deemed necessary for the proposed models.

### 3.2. Performance Evaluation on the Stratified Testing Set: Clean and Equivocal Test Cases

Table 5 displays the performances of the various models on the stratified testing set described in Section 2.7. The test cases were categorized based on their uncertainty levels, as defined in Section 2.3, and were evaluated separately using different models. The upper panel (a) presents the results for the 477 CXRs with uncertainty levels smaller than two, which were referred to as clean test cases. The lower panel (b) shows the results for the 528 cases with higher uncertainty, which were denoted as equivocal test cases. In line with the findings discussed in Section 3.1, the models incorporating the thresholding mechanism demonstrated superior performance compared to those without it. Therefore, the table includes only the models utilizing the thresholding mechanism, along with the Linear Probing, Fine Tuning, and Confusion Estimation approaches, for a comprehensive comparison.

When evaluating the clean test cases, the proposed model with a Score Encoding approach demonstrated the highest performance across all the evaluated metrics. It achieved a precision of 0.921, an accuracy of 0.921, an AUPRC of 0.971, an AUROC of 0.973, a sensitivity of 0.92, a specificity of 0.921, an F1 score of 0.921, and an MCC of 0.840. The model that utilized the Separate Encoding method achieved the same level of performance, while using the Combined Encoding approach showed slightly inferior results. Comparing the proposed models with the Linear Probing and Fine Tuning methodologies, the proposed models showed a 1% increase in the AUROC and AUPRC, an around 7% increase in MCC, and a 3–4% improvement on the other metrics. In addition, the Linear Probing and Fine Tuning approaches exhibited similar levels of performance, with differences of less than 1%. The Confusion Estimation model performed on par with or slightly worse than TRAM with thresholding. For the TRAM models that used the thresholding mechanism, those utilizing the Separate Encoding method achieved the highest performance on the clean test cases compared to the other two ways of encoding. However, their overall performance was 1% to 2% lower than that of the proposed models across most metrics, and they exhibited higher standard deviations.

When considering the equivocal test cases, the Confusion Estimation approach achieved the highest AUPRC at 0.737, as well as an AUROC of 0.728. A TRAM approach with thresholding and the Separate Encoding methodology demonstrated the best performance in terms of accuracy, sensitivity, F1 score, and MCC. The proposed model with a Score Encoding approach achieved the highest precision and specificity. Overall, the proposed model did not consistently outperform the other models in cases of label ambiguity.

It is important to note that, in the context of equivocal test cases that are potentially being assigned with incorrect labels, higher values could indicate overfitting to the noisy labels. Therefore, we were more concerned with the performances on the clean test cases as they can provide a more accurate reflection of how each model performs. Interestingly, although the Confusion Estimation model achieved the best AUPRC overall, as shown in Table 4, its performances on the clean test cases were worse than the proposed method. Another interesting observation was that the performances on the equivocal test cases were generally worse compared to the clean cases, whereby a decrease of approximately 25% was exhibited. Additionally, the standard deviations were generally larger for the equivocal test cases. These findings indicated that the presence of uncertainty in test cases can significantly impact model performance and increase variability in the results.

### 3.3. Qualitative Results Using the Score-CAM Approach on Test Images

**Figure 5:**
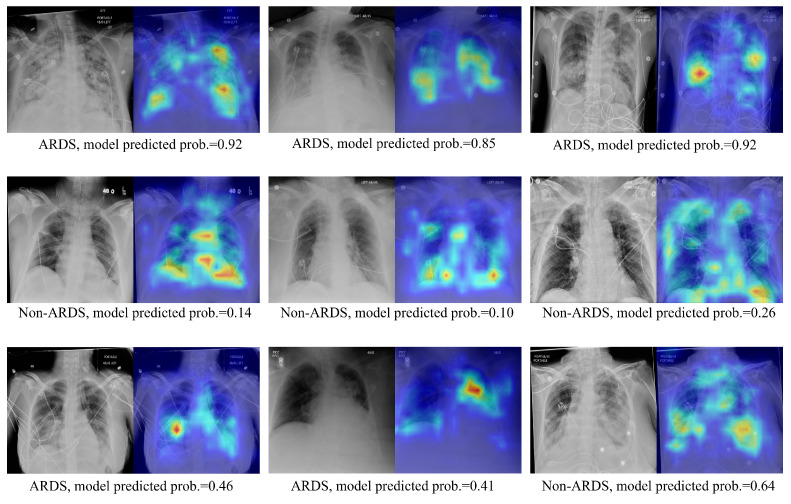
Image pair featuring the original CXR alongside ScoreCAM heatmap-overlaid CXRs. The text below each image pair indicates the ground truth ARDS label and the probability of ARDS as predicted by the target model.

Visualizations of the CXR images and their corresponding ScoreCAM heat maps from the proposed method are presented in Figure 5. The highlighted areas signify regions of interest during the model’s decision-making process for relevant classes. The upper panel exhibits the true positive cases, the middle panel displays true negative predictions, and the lower panel showcases highlighted regions when the model produces false positive or false negative predictions. In instances of true positive predictions, the areas of interest predominantly focus on lung regions that exhibit consolidations. True negative predictions see the model emphasizing the overall lung region and its corners. For incorrectly predicted cases, the model either concentrates on the region of interest but fails to make the correct prediction or is unable to highlight the correct region of interest. In addition, despite the existence of various medical devices, the model has not erroneously emphasized them in its predictions. This indicates the model’s robustness against confounding structures.

## 4. Discussion

CXRs are commonly used for ARDS diagnosis, but their interpretation can be challenging and subjective. Previous studies have utilized traditional machine learning and deep learning approaches to detect ARDS from CXR images. However, these approaches have not adequately addressed label uncertainty and noise, which can affect model performance. In this study, inspired by the TRAM network, we proposed a deep learning model that leverages label uncertainty from multiple annotators as privileged information to improve ARDS detection in CXR images. We introduced three different encoding methods and a simple, but effective, measure of uncertainty to supply the model with privileged information when necessary. Additionally, we applied ordinal regression to the privileged branch of the model to encourage knowledge transfer across branches. Our proposed model achieved an AUROC of 0.873, an AUPRC of 0.868, an F1 score of 0.797, and an MCC of 0.591 on the test examples. Moreover, it achieved an AUROC of 0.973, an AUPRC of 0.971, an F1 score of 0.921, and an MCC of 0.84 on cases with more certain and cleaner labels; meanwhile, fine tuning the encoder only produced an AUROC of 0.956, an AUPRC of 0.956, an F1 score of 0.890, and an MCC of 0.773. In comparison to the two previous studies of [8,9], which primarily focused on developing models for ARDS detection and used ARDS datasets that have similar attributes to ours, our work specifically addresses the challenge of leveraging multi-annotator label uncertainty to enhance performance.

This study also presents findings and insights regarding the TRAM mechanism. As the first application of the TRAM method in medical image analysis, our experiments highlighted its utility in the identification of ARDS from CXR images, while also validating previous findings [28] that excessive privileged information can hinder model generalizability. Specifically, we demonstrated the critical role of the thresholding mechanism in the success of our proposed model. Although we used a median value of the uncertainty measurements in the training set as the determined threshold, this value can be regarded as a hyperparameter when using the proposed method on other datasets. In Appendix B, we explore the impact of different threshold values on cross-validation and testing performance. It was observed that increasing the threshold from 0 to 4 in increments of 0.5 initially enhances testing performance but subsequently leads to a decline. Another interesting observation was that, despite the maximum measurement of uncertainty being 3.96 and that applying a threshold of 4 results in the TRAM network receiving no privileged information, the results presented in Table 4, Appendix B, and Table A1 showed that using a threshold of 4 outperforms both the approach of supplying extensive privileged information without thresholding and fine tuning the network. This performance improvement when using the TRAM mechanism, even in the absence of additional information, could be attributed to two key factors. First, the privileged prediction head in our experimental setup exhibits a stronger learning capability. While the base network employs a single-layer prediction head, the privileged branch incorporates a larger prediction head with enhanced learning capacity. Consequently, knowledge from the prediction head, rather than privileged information itself, can be learned and transferred to the base network. Second, the presence of two branches and the stop-gradient operation in a TRAM approach may contribute to mitigating overfitting tendencies. We observed more stable training loss behavior and less overfitting when employing the TRAM-based network compared to fine tuning.

While fine tuning a supervised pretrained feature encoder is the most common approach for transfer learning in medical imaging tasks, recent studies [35,36,37,38] have explored the effectiveness of self-supervised pretraining in CXR image analysis; in addition, some studies [35,38] have shown that self-supervised pretrained feature encoders generate more informative embeddings compared to their supervised counterparts. To assess if our proposed model consistently achieves a superior performance with different pretrained encoders, and to explore whether self-supervised pretraining can yield better encoders for ARDS detection than their supervised counterparts, we conducted additional experiments using Boost Your Own Latent (BYOL) [39] and DINO [40] pretrained encoders. Detailed information regarding the background, training protocol, and results of these experiments can be found in Appendix C. In summary, our findings demonstrate that utilizing DINO pretrained encoders can enhance the performance of ARDS detection compared to supervised pretrained encoders. Moreover, while the quality of the pretrained encoder and its architecture are crucial factors influencing downstream fine tuning performance, the methods proposed in this paper consistently yielded matching or superior test performance compared to other baselines, regardless of the specific pretrained encoder employed.

Our work has certain limitations that should be acknowledged, with the primary limitation relating to interpretability. Firstly, the proposed models do not provide insights into how different annotators contribute to label noise or how their annotations impact the final results, whereas the Confusion Estimation model [17] offers a potential solution by estimating the skill level of each annotator based on their confusion matrix’s average diagonal elements. Furthermore, the encoding methods that performed best in testing for the proposed models favored the Score Encoding and Separate Encoding approaches, which do not rely on the correspondence between the scale and its annotator. This observation suggests that the model’s performance may not be dependent on this correspondence, and the mechanism by which it utilizes multi-annotator information still lacks interpretability.

In a recent study by Farzaneh et al. [41], who investigated collaborative strategies between physicians and an artificial intelligence (AI) model in ARDS diagnosis, it was discovered that AI and physician expertise complement each other. The AI model exhibited higher and more consistent accuracy on less challenging CXRs, while physicians demonstrated higher accuracy on difficult CXRs. These findings endorse the strategy of having the AI model review CXRs initially and involve clinicians when uncertainty arises, thus highlighting the need to identify uncertain cases that will guide the future direction of our work. Specifically, our focus will be on enhancing the interpretability of the uncertainty level associated with each case and integrating strategies to handle noisy labels at both the annotator and sample levels. By doing so, we aim to further support the early identification of ARDS to enhance evidence-based care.

## 5. Conclusions

In conclusion, this study introduces a DL model that effectively utilizes label uncertainty from multiple annotators as privileged information to improve the detection of ARDS in CXR images. By employing ordinal regression on the privileged branch and implementing a threshold mechanism, we observed improvements in the testing performance across various evaluation metrics, including AUROC, AUPRC, accuracy, sensitivity, specificity, precision, and F1 score, while also achieving lower standard deviations. These results highlight the critical role of considering label uncertainty and noise in ARDS diagnosis and reinforce the value of incorporating a thresholding mechanism in a TRAM-based approach. The advancements made in this work have the potential to enhance patient care and decision-making processes in ARDS by providing healthcare professionals with accurate and dependable support.

## Figures and Tables

**Table 1:** Number of patients and CXR images (ARDS and non-ARDS) in the training and testing sets. All numbers shown are counts.

	Patient	ARDS CXRs	Non-ARDS CXRs	Total CXRs
**Train**	333	606	1444	2050
**Test**	167	327	678	1005
**Total**	500	933	2122	3055

**Table 2:** Demographics of the patients included in this study.

	Patients (N)	Age (yrs)
**Male**	309	57.16 ± 16.72
**Female**	191	58.46 ± 15.71
**Total**	500	57.65 ± 16.32

**Table 3:** Summary statistics of the measurement of uncertainty on the training and test sets.

Uncertainty *D*	Mean	Std.	Min.	Q1 ^*a*^	Median	Q3 ^*b*^	Max.
Training	1.95	1.29	0.00	1.00	2.00	3.25	3.92
Testing	1.86	1.23	0.00	1.00	2.00	3.22	3.83

^*a*^ 25th percentile and ^*b*^ 75th percentile.

**Table 4:** Test performances across different models with mean-aggregated labels for the (a) baseline and (b) proposed methods.

(a). Baselines	Precision	Accuracy	AUPRC	AUROC	Sensitivity	Specificity	F1 Score	MCC
Linear Probing	0.768 ± 0.006	0.771 ± 0.006	0.838 ± 0.010	0.850 ± 0.008	0.776 ± 0.007	0.765 ± 0.006	0.772 ± 0.006	0.541 ± 0.012
Fine Tuning	0.771 ± 0.015	0.772 ± 0.010	0.855 ± 0.008	0.856 ± 0.012	0.775 ± 0.014	0.769 ± 0.009	0.773 ± 0.012	0.545 ± 0.014
Confusion Estimation [17]	0.785 ± 0.010	0.788 ± 0.009	**0.870 ± 0.001**	0.871 ± 0.001	0.794 ± 0.008	0.782 ± 0.010	0.789 ± 0.009	0.576 ± 0.018
TRAM [18] + Score. E.	0.763 ± 0.016	0.766 ± 0.016	0.835 ± 0.018	0.842 ± 0.017	0.773 ± 0.015	0.760 ± 0.016	0.768 ± 0.015	0.533 ± 0.031
TRAM [18] + Separ. E.	0.758 ± 0.012	0.762 ± 0.013	0.836 ± 0.018	0.842 ± 0.015	0.767 ± 0.013	0.756 ± 0.012	0.763 ± 0.013	0.523 ± 0.025
TRAM [18] + Comb. E.	0.764 ± 0.014	0.770 ± 0.014	0.834 ± 0.015	0.846 ± 0.012	0.781 ± 0.013	0.758 ± 0.014	0.772 ± 0.014	0.539 ± 0.027
TRAM w/Thresh. + Score. E.	0.785 ± 0.013	0.788 ± 0.014	0.860 ± 0.011	0.866 ± 0.009	0.795 ± 0.016	0.782 ± 0.013	0.790 ± 0.015	0.577 ± 0.029
TRAM w/Thresh. + Separ. E.	**0.792 ± 0.011**	**0.796 ± 0.012**	0.866 ± 0.016	**0.872 ± 0.011**	**0.802 ± 0.014**	**0.789 ± 0.010**	**0.797 ± 0.012**	**0.591 ± 0.024**
TRAM w/Thresh. + Comb. E.	0.786 ± 0.031	0.789 ± 0.033	0.859 ± 0.020	0.865 ± 0.021	0.796 ± 0.034	0.783 ± 0.031	0.791 ± 0.033	0.579 ± 0.065
TRAM w/Ord. Reg. + Score. E.	0.790 ± 0.007	0.790 ± 0.007	0.852 ± 0.017	0.861 ± 0.014	0.789 ± 0.008	0.790 ± 0.007	0.789 ± 0.007	0.579 ± 0.014
TRAM w/Ord. Reg. + Separ. E.	0.792 ± 0.009	0.792 ± 0.009	0.852 ± 0.016	0.860 ± 0.013	0.791 ± 0.009	0.792 ± 0.009	0.791 ± 0.009	0.583 ± 0.018
TRAM w/Ord. Reg. + Comb. E.	0.780 ± 0.014	0.780 ± 0.013	0.850 ± 0.014	0.858 ± 0.012	0.779 ± 0.012	0.780 ± 0.015	0.780 ± 0.013	0.560 ± 0.027
**(b). Proposed Models**	**Precision**	**Accuracy**	**AUPRC**	**AUROC**	**Sensitivity**	**Specificity**	**F1 Score**	**MCC**
Proposed + Score. E.	**0.798 ± 0.007**	**0.797 ± 0.006**	**0.868 ± 0.012**	**0.873 ± 0.010**	**0.796 ± 0.006**	**0.798 ± 0.007**	**0.797 ± 0.006**	**0.594 ± 0.012**
Proposed + Separ. E.	0.796 ± 0.008	0.795 ± 0.007	0.864 ± 0.015	0.871 ± 0.012	0.793 ± 0.006	0.796 ± 0.008	0.794 ± 0.007	0.589 ± 0.014
Proposed + Comb. E.	0.789 ± 0.003	0.789 ± 0.003	0.863 ± 0.014	0.868 ± 0.010	0.788 ± 0.004	0.789 ± 0.003	0.789 ± 0.003	0.577 ± 0.006

Notes: The best performances under different metrics are highlighted in **bold** for (**a**) baseline models and (**b**) proposed models, separately.

**Table 5:** The performances across different models on the stratified test set.

(a). Uncertainty ∈ [0, 2), *n* = 477	Precision	Accuracy	AUPRC	AUROC	Sensitivity	Specificity	F1 Score	MCC
Linear Probing	0.882 ± 0.009	0.886 ± 0.007	0.955 ± 0.006	0.958 ± 0.005	0.892 ± 0.005	0.881 ± 0.009	0.887 ± 0.007	0.773 ± 0.015
Fine Tuning	0.887 ± 0.006	0.889 ± 0.007	0.956 ± 0.003	0.956 ± 0.004	0.892 ± 0.008	0.887 ± 0.006	0.890 ± 0.007	0.778 ± 0.013
Confusion Estimation [17]	0.900 ± 0.004	0.903 ± 0.005	0.965 ± 0.002	0.965 ± 0.002	0.907 ± 0.007	0.899 ± 0.004	0.904 ± 0.005	0.806 ± 0.010
TRAM w/Thresh. + Score. E.	0.908 ± 0.008	0.911 ± 0.008	0.961 ± 0.009	0.965 ± 0.008	0.914 ± 0.008	0.907 ± 0.008	0.911 ± 0.008	0.821 ± 0.016
TRAM w/Thresh. + Separ. E.	0.911 ± 0.007	0.914 ± 0.007	0.966 ± 0.011	0.969 ± 0.007	0.918 ± 0.008	0.910 ± 0.007	0.914 ± 0.007	0.827 ± 0.015
TRAM w/Thresh. + Comb. E.	0.902 ± 0.035	0.905 ± 0.035	0.962 ± 0.012	0.964 ± 0.012	0.909 ± 0.034	0.901 ± 0.035	0.906 ± 0.034	0.811 ± 0.069
Proposed + Score. E.	**0.921 ± 0.006**	**0.921 ± 0.005**	**0.971 ± 0.005**	**0.973 ± 0.003**	**0.920 ± 0.004**	**0.921 ± 0.006**	**0.921 ± 0.005**	**0.840 ± 0.009**
Proposed + Separ. E.	0.920 ± 0.008	0.920 ± 0.008	0.969 ± 0.007	0.972 ± 0.005	0.919 ± 0.009	0.920 ± 0.008	0.920 ± 0.008	0.839 ± 0.017
Proposed + Comb. E.	0.915 ± 0.003	0.915 ± 0.004	0.969 ± 0.005	0.971 ± 0.003	0.915 ± 0.004	0.915 ± 0.003	0.915 ± 0.004	0.830 ± 0.008
**(b). Uncertainty ∈ [2, 4],** ***n*** **= 528**	**Precision**	**Accuracy**	**AUPRC**	**AUROC**	**Sensitivity**	**Specificity**	**F1 Score**	**MCC**
Linear Probing	0.664 ± 0.005	0.666 ± 0.006	0.693 ± 0.008	0.724 ± 0.008	0.672 ± 0.008	0.660 ± 0.003	0.668 ± 0.006	0.332 ± 0.011
Fine Tuning	0.666 ± 0.013	0.667 ± 0.013	0.720 ± 0.013	0.731 ± 0.010	0.670 ± 0.014	0.664 ± 0.013	0.668 ± 0.014	0.334 ± 0.027
Confusion Estimation [17]	0.681 ± 0.015	0.684 ± 0.013	**0.737 ± 0.006**	**0.748 ± 0.003**	0.691 ± 0.009	0.677 ± 0.017	0.686 ± 0.012	0.368 ± 0.027
TRAM w/Thresh. + Score. E.	0.675 ± 0.019	0.678 ± 0.021	0.715 ± 0.015	0.732 ± 0.011	0.688 ± 0.026	0.669 ± 0.017	0.681 ± 0.022	0.356 ± 0.042
TRAM w/Thresh. + Separ. E.	0.685 ± 0.015	**0.689 ± 0.016**	0.718 ± 0.022	0.738 ± 0.018	**0.698 ± 0.019**	0.680 ± 0.014	**0.691 ± 0.017**	**0.378 ± 0.033**
TRAM w/Thresh. + Comb. E.	0.681 ± 0.031	0.685 ± 0.033	0.715 ± 0.021	0.736 ± 0.026	0.694 ± 0.035	0.676 ± 0.030	0.687 ± 0.033	0.369 ± 0.065
Proposed + Score. E.	**0.686 ± 0.009**	0.686 ± 0.009	0.715 ± 0.017	0.737 ± 0.018	0.684 ± 0.009	**0.687 ± 0.009**	0.685 ± 0.009	0.371 ± 0.017
Proposed + Separ. E.	0.683 ± 0.008	0.682 ± 0.007	0.709 ± 0.020	0.733 ± 0.018	0.679 ± 0.006	0.684 ± 0.009	0.681 ± 0.007	0.363 ± 0.014
Proposed + Comb. E.	0.675 ± 0.004	0.675 ± 0.004	0.712 ± 0.025	0.731 ± 0.018	0.673 ± 0.004	0.676 ± 0.005	0.674 ± 0.004	0.349 ± 0.008

Notes: The best performances under different metrics are highlighted in **bold** for test results in panels (**a**) and (**b**), separately.

**Figure A1:**
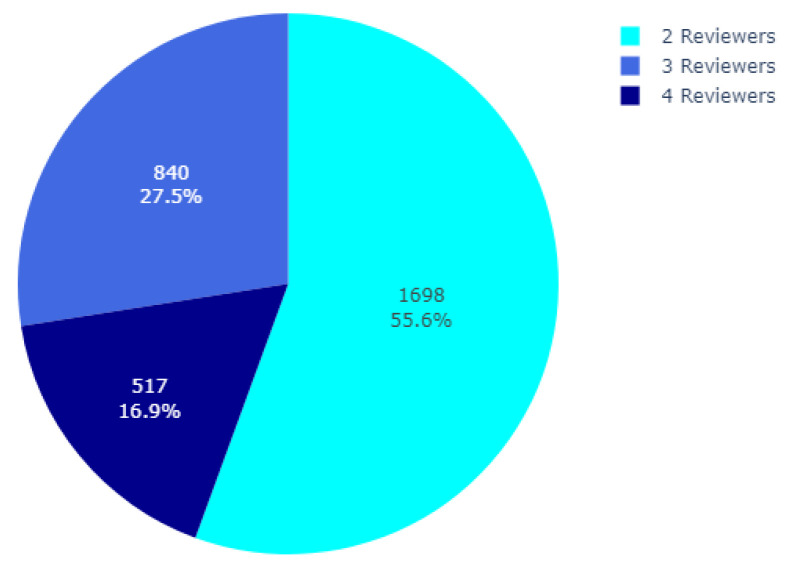
The distribution of the number of reviewers on each image.

**Figure A2:**
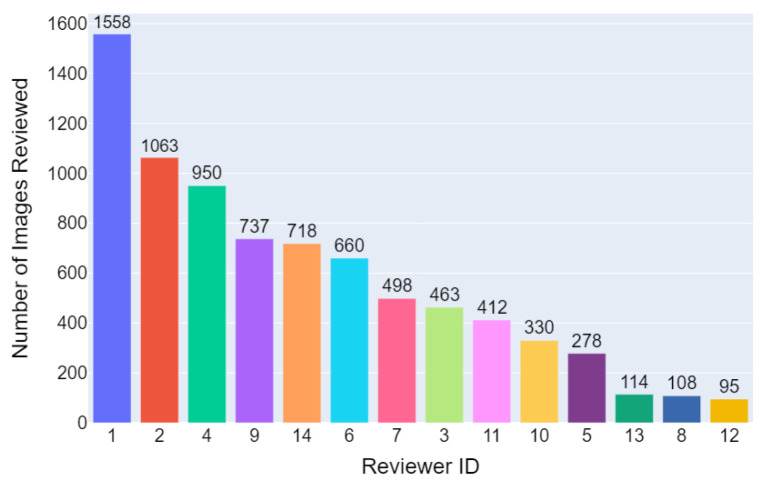
The distribution of the total number of images reviewed by each reviewer.

## Data Availability

The datasets generated and/or analyzed during the current study were collected at Michigan Medicine. The University of Michigan’s Innovation Partnerships (UMIP) unit will handle potential charges/arrangements of the use of data by external entities, using such methods as material transfer agreements. Please contact UMIP (innovationpartnerships@umich.edu) for data inquiries. The underlying code for this study is available in Github and can be accessed via this link https://github.com/kayvanlabs/Uncertainty-TRAM (accessed on 27 January 2024).

## Appendix A. Reviewer Assignment and Review Distribution

Figure A1 depicts the distribution of the number of reviewers assigned to each image. Approximately 55.6% (1698) of the images were reviewed by two independent reviewers, while 27.5% (840) were reviewed by three reviewers. The remaining 16.9% (517) of the images underwent review by four reviewers. And, based on Figure A2, the reviewer with the highest number of review cases examined 1558 chest X-ray images, while the reviewer with the fewest number of images reviewed examined 95 records. Reviewers 1, 2, 3, 9, 14, and 6 individually reviewed over 500 images, thereby collectively accounting for more than 50% of the total reviews.

## Appendix B. Impact of Threshold Levels on the Validation and Testing Results

Table A1 presents the results of the proposed models with the Scale Encoding approach as an example, which also illustrates the impact of threshold levels on validation and testing outcomes. As the threshold level increased from 0 to 4 with a 0.5 basis, the validation performance initially improved and then declined. The lowest validation loss and highest F1 score were achieved when thresholding at 3. However, these metrics exhibited large standard deviations. Considering this trend, a threshold of 2.5 could be an optimal value as it demonstrates a similar averaged metric and lower standard deviation. The best validation AUROC and AUPRC values were also achieved at the threshold of 2.5. The testing performance exhibited a similar trend of initially increasing and then decreasing. The best precision, sensitivity, specificity, and F1 score were achieved with a threshold of 1.5, while the best AUPRC and AUROC values were obtained when thresholding at 2.5. These findings suggest that providing the model with more privileged information relative to non-privileged information initially enhances performance, but beyond a certain threshold, the performance begins to deteriorate. Furthermore, the results indicate that the threshold used in our experiments may not be the optimal choice, but they can be deemed valid and appropriate within the context of our study.

**Table A1:** The validation and testing outcomes for the proposed model using the Scale Encoding approach with different thresholds.

	Validation Outcomes				Test Outcomes					
	**Loss**	**AUROC**	**AUPRC**	**F1 Score**	**Precision**	**AUPRC**	**AUROC**	**Sensitivity**	**Specificity**	**F1 Score**
w/o Thred.	0.468 ± 0.003	0.952 ± 0.010	0.946 ± 0.010	0.884 ± 0.007	0.790 ± 0.007	0.852 ± 0.017	0.861 ± 0.014	0.789 ± 0.008	0.790 ± 0.007	0.789 ± 0.007
Thred. 0.5	0.449 ± 0.006	0.958 ± 0.010	0.954 ± 0.013	0.901 ± 0.015	0.792 ± 0.007	0.857 ± 0.013	0.867 ± 0.009	0.791 ± 0.006	0.792 ± 0.008	0.791 ± 0.006
Thred. 1.0	0.445 ± 0.003	0.960 ± 0.009	0.956 ± 0.011	0.902 ± 0.013	0.796 ± 0.006	0.863 ± 0.009	0.870 ± 0.008	0.793 ± 0.007	0.797 ± 0.005	0.794 ± 0.006
Thred. 1.5	0.442 ± 0.008	0.958 ± 0.013	0.953 ± 0.016	0.899 ± 0.012	**0.797 ± 0.006**	0.865 ± 0.012	0.871 ± 0.010	**0.796 ± 0.007**	**0.798 ± 0.005**	**0.797 ± 0.006**
Thred. 2.0	0.439 ± 0.008	0.960 ± 0.011	0.955 ± 0.014	0.901 ± 0.015	0.796 ± 0.007	0.868 ± 0.012	0.873 ± 0.010	0.795 ± 0.006	0.796 ± 0.007	0.795 ± 0.006
Thred. 2.5	0.435 ± 0.007	**0.961 ± 0.011**	**0.956 ± 0.014**	0.903 ± 0.014	0.794 ± 0.005	**0.871 ± 0.011**	**0.875 ± 0.010**	0.793 ± 0.005	0.795 ± 0.005	0.794 ± 0.005
Thred. 3.0	0.437 ± 0.010	0.960 ± 0.013	0.955 ± 0.016	0.901 ± 0.014	0.792 ± 0.007	0.869 ± 0.010	0.873 ± 0.009	0.790 ± 0.005	0.793 ± 0.008	0.791 ± 0.006
Thred. 3.5	**0.433 ± 0.019**	0.959 ± 0.019	0.954 ± 0.023	**0.904 ± 0.022**	0.794 ± 0.010	0.869 ± 0.014	0.873 ± 0.011	0.791 ± 0.009	0.795 ± 0.011	0.793 ± 0.010
Thred. 4.0	0.443 ± 0.014	0.958 ± 0.017	0.952 ± 0.024	0.903 ± 0.016	0.789 ± 0.004	0.864 ± 0.013	0.870 ± 0.010	0.787 ± 0.004	0.790 ± 0.005	0.788 ± 0.004

Notes: The best performances under different metrics are highlighted in **bold**.

## Appendix C. Results with Self-Supervised Pretrained Encoders

Self-supervised learning (SSL) [42] is a powerful approach that utilizes large unlabeled datasets to train models in a task-agnostic manner. Unlike traditional supervised learning, which relies on labeled data, SSL derives its supervisory signal from the inherent structure and patterns within the data themselves. SSL can be broadly categorized into two main types: contrastive and non-contrastive methods. Both aim to capture meaningful and discriminative features that are valuable for downstream tasks. Contrastive SSL involves training the model to bring similar images closer together in the embedding space while pushing dissimilar images apart. By optimizing the embeddings based on similarity, the model learns to extract informative visual representations. On the other hand, non-contrastive SSL, which is represented by self-distillation methods, encourages the model to learn consistent embeddings from different views of the same image. This process of learning from the model’s own predictions fosters the development of robust and informative embeddings. Notable works in the realm of non-contrastive SSL include BYOL [39] and DINO [40].

BYOL utilizes two networks, the “online” and the “target”, where each network is presented with a different view of the same image through image transformations. During training, the online network is updated using the gradient descent method, and it is based on its predictions of the representation for the differently augmented view. At the same time, the target network, serving as a reference, is updated using exponential moving average updates of the weights from the online network. This process encourages the encoder to learn meaningful embeddings that are robust to different data augmentations, thus enabling it to capture useful features in the desired image domain. In DINO, two networks with identical Vision Transformer (ViT) models are employed: one acts as the student network and the other as the teacher network. These networks receive input from two sets of views obtained by cropping the same image, thus allowing them to capture the semantic relationship between the local and global crops through self-attention mechanisms. Both BYOL and DINO have shown promising results in learning powerful visual representations without the need for manual annotations and contrastive examples, and they have recently been applied as pretraining methods for chest disease classification in CXRs [35,36,37].

In the following section, we provide details of the experiments conducted using BYOL and DINO pretrained encoders. Unless stated otherwise, the implementation details closely follow those described in Section 2.

### Appendix C.1. BYOL Pretrained Encoder

#### Appendix C.1.1. Dataset

The CheXpert [26] dataset consists of 224,316 chest radiographs from 65,240 patients, with both frontal and lateral projections available. For the self-supervised pre-training in this study, only frontal projections with either anteroposterior (AP) or posteroanterior (PA) chest views from the original training set were retained, thus resulting in n = 191,010 chest X-rays from N = 64,534 patients. The dataset was then split patient-wise, with 80% (N = 51,628, n = 153,813) of the patients assigned to the training set and the remaining 20% (N = 12,906, n = 37,197) assigned to the validation.

#### Appendix C.1.2. The Pre-Training Protocol

The ResNet50 network was employed as the encoders and was pretrained using the BYOL framework. The projectors and the predictor followed the original BYOL implementation, in which the linear layer comprised an output size of 4096, a batch normalization layer, a ReLU activation function, and a linear layer with an output size of 256. By referencing [43], we used a random resized cropping of scale (3/4, 4/3), as well as random contrast and brightness adjustments as the augmentation strategy for BYOL training. The image augmentation was carried out using the Kornia library [44].

In addition, the pre-training utilized a batch size of 128, where the SGD optimizer was employed with a learning rate of 0.03, a momentum of 0.9, and a weight decay of 0.0004. To facilitate the training process, a linear warm-up cosine annealing scheduler was applied for the initial 5 epochs. Subsequently, the training continues for a total of 20 epochs, and the epoch that yielded the lowest validation loss was selected for the downstream task.

The implementation of BYOL was based on a publicly available code repository (https://github.com/lucidrains/byol-pytorch (accessed on 23 April 2023)), which was made avalaible by [39]. However, certain modifications were made by removing the hook registration while leaving the remaining code unchanged.

### Appendix C.2. DINO Pretrained Encoder

For the DINO pretrained encoder, we utilized the implementation from the paper of [37], and the weights were obtained from the official code repository (https://github.com/sangjoon-park/AI-Can-Self-Evolve (accessed on 23 April 2023)). According to abovementioned paper, the encoder architecture was a small ViT model with 12 layers and 6 heads, which used a patch size of 8 × 8. The pretraining dataset was still CheXpert. During the pretraining process, an Adam optimizer with a learning rate of 0.0001 was utilized. The encoder was pretrained for a total of 5 epochs, and a step decay scheduler was employed. The batch size for the pretraining phase was set to 16. In terms of data augmentation, weak transformations such as random flipping, rotation, and translation were applied to enhance the training diversity. For more detailed information on the image preprocessing and implementation of the DINO method, we recommend referring to the method section of the original paper [37].

### Appendix C.3. Results

Table A2 presents the results obtained when using a BYOL pre-trained encoder. Among all the listed models, the proposed model with a Scale Encoding approach achieved the highest precision, accuracy, AUROC, specificity, and F1 score, while the best AUPRC and sensitivity values were attained by the proposed model with a Separate Encoding method. TRAM with a Threshold Mechanism approach demonstrated similar levels of performance as one with the Fine Tuning methodology, except for it producing a higher sensitivity, F1 score, and lower specificity. The Confusion Estimation method achieved slightly lower performance than the proposed models. On the other hand, the Linear Probing approach performed significantly worse compared to the other models.

**Table A2:** Testing performance with a BYOL pretrained encoder on all test cases and clean test cases.

(a). All Test Cases, *n* = 1005	Precision	Accuracy	AUPRC	AUROC	Sensitivity	Specificity	F1 Score
Linear Probing	0.670 ± 0.006	0.694 ± 0.008	0.764 ± 0.005	0.770 ± 0.005	0.766 ± 0.018	0.622 ± 0.011	0.714 ± 0.009
Fine-tuning	0.743 ± 0.007	0.754 ± 0.007	0.835 ± 0.010	0.838 ± 0.009	0.777 ± 0.010	0.731 ± 0.008	0.759 ± 0.007
Confusion Estimation	0.747 ± 0.007	0.762 ± 0.007	0.840 ± 0.011	0.841 ± 0.010	0.794 ± 0.008	0.730 ± 0.007	0.770 ± 0.007
TRAM w/Thresh. + Scale. E.	0.727 ± 0.009	0.750 ± 0.010	0.833 ± 0.007	0.836 ± 0.006	0.799 ± 0.011	0.700 ± 0.010	0.761 ± 0.010
TRAM w/Thresh. + Separ. E.	0.732 ± 0.009	0.753 ± 0.009	0.837 ± 0.005	0.839 ± 0.005	0.800 ± 0.012	0.707 ± 0.011	0.764 ± 0.009
TRAM w/Thresh. + Comb. E.	0.741 ± 0.005	0.755 ± 0.003	0.839 ± 0.005	0.842 ± 0.002	0.785 ± 0.010	0.725 ± 0.010	0.762 ± 0.004
Proposed + Scale. E.	**0.755 ± 0.009**	**0.770 ± 0.007**	0.838 ± 0.008	**0.850 ± 0.004**	0.798 ± 0.003	**0.741 ± 0.012**	**0.776 ± 0.006**
Proposed + Separ. E.	0.739 ± 0.005	0.761 ± 0.006	**0.843 ± 0.009**	0.849 ± 0.006	**0.808 ± 0.009**	0.715 ± 0.005	0.772 ± 0.007
Proposed + Comb. E.	0.749 ± 0.002	0.766 ± 0.001	0.838 ± 0.010	0.848 ± 0.005	0.801 ± 0.003	0.731 ± 0.004	0.774 ± 0.001
**(b). Clean Test Cases,** ***n*** **= 477**	**Precision**	**Accuracy**	**AUPRC**	**AUROC**	**Sensitivity**	**Specificity**	**F1 Score**
Linear Probing	0.733 ± 0.013	0.765 ± 0.016	0.857 ± 0.006	0.858 ± 0.008	0.834 ± 0.027	0.695 ± 0.017	0.780 ± 0.017
Fine Tuning	0.854 ± 0.009	0.867 ± 0.007	0.943 ± 0.003	0.944 ± 0.005	0.885 ± 0.004	0.849 ± 0.010	0.869 ± 0.006
Confusion Estimation	0.872 ± 0.010	0.886 ± 0.007	**0.952 ± 0.006**	0.953 ± 0.007	0.906 ± 0.003	0.867 ± 0.012	0.889 ± 0.006
TRAM w/Thresh. + Scale. E.	0.819 ± 0.018	0.846 ± 0.019	0.929 ± 0.015	0.932 ± 0.012	0.887 ± 0.018	0.804 ± 0.020	0.852 ± 0.018
TRAM w/Thresh. + Separ. E.	0.831 ± 0.016	0.855 ± 0.016	0.930 ± 0.015	0.933 ± 0.012	0.891 ± 0.018	0.818 ± 0.018	0.860 ± 0.016
TRAM w/Thresh. + Comb. E.	0.839 ± 0.009	0.853 ± 0.010	0.936 ± 0.005	0.938 ± 0.003	0.873 ± 0.013	0.832 ± 0.010	0.855 ± 0.010
Proposed + Scale. E.	**0.873 ± 0.007**	**0.887 ± 0.007**	0.951 ± 0.003	**0.954 ± 0.001**	**0.906 ± 0.010**	**0.868 ± 0.008**	**0.889 ± 0.007**
Proposed + Separ. E.	0.849 ± 0.001	0.872 ± 0.006	0.948 ± 0.007	0.949 ± 0.004	0.905 ± 0.014	0.839 ± 0.002	0.876 ± 0.007
Proposed + Comb. E.	0.860 ± 0.008	0.878 ± 0.010	0.948 ± 0.005	0.949 ± 0.004	0.904 ± 0.013	0.853 ± 0.009	0.881 ± 0.010

Notes: The best performances under different metrics are highlighted in **bold** for test results in panels (**a**) and (**b**), separately.

When evaluating on the clean cases, the proposed method with a Scale Encoding methodology demonstrated the highest values on almost all metrics, while the Confusion Estimation approach also achieved the same level of performance, but also exhibited the best AUPRC. The TRAM with thresholding showed a 2–4% lower performance compared to the proposed methods and the Confusion Estimation approach across almost all metrics. And it did not perform as well as the Fine Tuning method in terms of overall performance.

Table A3 presents the results of the DINO pretrained encoders. The proposed methods with separate encoding achieved the highest accuracy, AUROC, and F1 score, while the Confusion Estimation appraoch obtained the optimal AUPRC, AUROC, sensitivity, and F1 score values. TRAM with thresholding and the Scale Encoding approach exhibited the best precision and specificity. However, there was no single model that consistently outperformed the others across all metrics. Overall, the Confusion Estimation method and the proposed methods with a Separate Encoding approach demonstrated the best performances. When tested on the clean cases, TRAM with thresholding and a Scale Encoding methodology achieved the highest precision and specificity. The proposed methods covered the optimal performance in the remaining metrics, thereby achieving an F1 score of 0.931 with the Separate Encoding method and an AUPRC of 0.974 together with an AUROC of 0.974 for the Combined Encoding approach. It is worth noting that TRAM with thresholding models tended to have higher precision and specificity but lower F1 scores and sensitivity compared to the proposed models and Confusion Estimation models.

**Table A3:** Testing performance with a DINO pretrained encoder on all test cases and clean test cases.

All Test Cases, *n* = 1005	Precision	Accuracy	AUPRC	AUROC	Sensitivity	Specificity	F1 Score
Linear Probing	0.672 ± 0.000	0.672 ± 0.000	0.609 ± 0.002	0.682 ± 0.002	0.672 ± 0.000	0.672 ± 0.000	0.672 ± 0.000
Fine Tuning	0.792 ± 0.007	0.789 ± 0.004	0.871 ± 0.006	0.872 ± 0.004	**0.783 ± 0.002**	0.794 ± 0.010	0.788 ± 0.003
Confusion Estimation	0.802 ± 0.006	0.795 ± 0.003	**0.873 ± 0.004**	**0.873 ± 0.003**	**0.783 ± 0.005**	0.806 ± 0.008	**0.792 ± 0.002**
TRAM w/Thresh. + Scale. E.	**0.829 ± 0.011**	0.784 ± 0.004	0.864 ± 0.002	0.866 ± 0.003	0.718 ± 0.023	**0.851 ± 0.016**	0.769 ± 0.009
TRAM w/Thresh. + Separ. E.	0.816 ± 0.010	0.790 ± 0.002	0.868 ± 0.001	0.869 ± 0.002	0.748 ± 0.015	0.832 ± 0.014	0.781 ± 0.004
TRAM w/Thresh. + Comb. E.	0.822 ± 0.005	0.792 ± 0.004	0.861 ± 0.003	0.867 ± 0.003	0.745 ± 0.015	0.838 ± 0.009	0.781 ± 0.007
Proposed + Scale. E.	0.813 ± 0.010	0.790 ± 0.007	0.869 ± 0.001	0.870 ± 0.003	0.753 ± 0.010	0.827 ± 0.011	0.782 ± 0.007
Proposed + Separ. E.	0.811 ± 0.014	**0.797 ± 0.004**	0.872 ± 0.003	**0.873 ± 0.005**	0.774 ± 0.013	0.819 ± 0.020	**0.792 ± 0.003**
Proposed + Comb. E.	0.824 ± 0.007	0.795 ± 0.003	0.868 ± 0.002	0.873 ± 0.004	0.750 ± 0.008	0.839 ± 0.008	0.785 ± 0.004
**(b). Clean Test Cases,** ***n*** **= 477**	**Precision**	**Accuracy**	**AUPRC**	**AUROC**	**Sensitivity**	**Specificity**	**F1 Score**
Linear Probing	0.711 ± 0.000	0.711 ± 0.000	0.613 ± 0.003	0.713 ± 0.002	0.711 ± 0.000	0.711 ± 0.000	0.711 ± 0.000
Fine Tuning	0.923 ± 0.009	0.922 ± 0.005	0.969 ± 0.003	0.971 ± 0.003	0.920 ± 0.004	0.923 ± 0.010	0.922 ± 0.004
Confusion Estimation	0.933 ± 0.008	0.927 ± 0.003	0.972 ± 0.002	0.973 ± 0.001	0.921 ± 0.004	0.934 ± 0.008	0.927 ± 0.003
TRAM w/Thresh. + Scale. E.	**0.946 ± 0.004**	0.922 ± 0.005	0.970 ± 0.002	0.970 ± 0.002	0.896 ± 0.015	**0.949 ± 0.005**	0.920 ± 0.006
TRAM w/Thresh. + Separ. E.	0.943 ± 0.005	0.929 ± 0.003	0.972 ± 0.002	0.972 ± 0.002	0.914 ± 0.006	0.945 ± 0.005	0.928 ± 0.003
TRAM w/Thresh. + Comb. E.	0.943 ± 0.003	0.924 ± 0.004	0.968 ± 0.002	0.969 ± 0.002	0.903 ± 0.011	0.945 ± 0.004	0.923 ± 0.005
Proposed + Scale. E.	0.937 ± 0.006	0.923 ± 0.005	0.970 ± 0.001	0.970 ± 0.002	0.908 ± 0.006	0.939 ± 0.007	0.922 ± 0.005
Proposed + Separ. E.	0.938 ± 0.004	**0.932 ± 0.002**	0.973 ± 0.002	0.973 ± 0.002	**0.925 ± 0.009**	0.939 ± 0.005	**0.931 ± 0.003**
Proposed + Comb. E.	0.943 ± 0.003	0.928 ± 0.003	**0.974 ± 0.001**	**0.974 ± 0.002**	0.912 ± 0.006	0.945 ± 0.003	0.927 ± 0.003

Notes: The best performances under different metrics are highlighted in **bold** for test results in panels (**a**) and (**b**), separately.
